# Evaluation of tumor response after stereotactic body radiation therapy for lung cancer: Role of 18F-fluorodeoxyglucose positron emission tomography/computed tomography

**Published:** 2020-10-06

**Authors:** Pino Alcantara, Beatriz Cabeza Martínez, Marta García García-Esquinas, Laura G. Belaústegui, Ana Bustos

**Affiliations:** ^1^Department of Radiation Oncology, Hospital Clinico San Carlos; ^2^Faculty of Medicine, Complutense University of Madrid; ^3^Department of Nuclear Medicine, Hospital Clinico San Carlos; ^4^Department of Radiology, Hospital Clinico San Carlos, Spain.

**Keywords:** lung cancer, positron emission tomography/computed tomography, recurrence, response assessment, stereotactic body radiation therapy

## Abstract

**Background:**

Early identification of patients who fail to lung stereotactic body radiation therapy (SBRT) is vital as they can benefit from salvage therapy. Main guidelines recommend computed tomography (CT) to assess response and use of 18F-fluorodeoxyglucose (^18^F-FDG) positron emission tomography (PET)/CT only when a local recurrence is suspected in CT. The pattern of radiation-induced lung injury caused by SBRT is different from changes seen after conventional radiation therapy in terms of extent, time of manifestation, and morphologic characteristics, and knowing this is crucial for proper monitoring of the tumor response. In certain cases, it may be difficult to differentiate response from progression or recurrence on CT and, in addition, some changes in CT take a long time to evolve before they are considered suspicious, making early diagnosis difficult. Metabolic changes often precede morphological changes, so ^18^F-FDG PET/CT quantitative and qualitative metabolic criteria can be useful in assessing early response and detecting relapses. However, the optimal practice for follow-up remains unclear and there is an active search for imaging markers for recurrent disease, including CT texture analysis, biomarker assays, new PET/CT isotopes, and magnetic resonance imaging.

**Aim:**

The aim of the study was to review the radiological changes that are objectified after pulmonary SBRT and the metabolic changes in ^1^F-FDG PET/CT, to assess the usefulness of following up patients with ^18^F-FDG PET/CT.

**Relevance for Patients:**

At present, the evaluation of response and diagnosis of relapse after SBRT are difficult and the incorporation of routine ^18^F-FDG PET/CT may have value in early diagnosis of relapse when the patient may still benefit from rescue treatment.

## 1. Introduction

In the past two decades, newer methods of planning and delivering radiation therapy have been developed, allowing safe and accurate administration of very high doses of radiation to a selected target. Stereotactic body radiation therapy (SBRT) is defined by the American College of Radiology and the American Society for Radiation Oncology as an “external beam radiation therapy method used to very precisely deliver a high dose of radiation to an extra-cranial target within the body, using either a single dose or a small number of fractions (hypofractionated)” [1]. In SBRT, the complex arrangement of multiple radiation beams converging on the target create a steep dose gradient with high and ablative doses to the tumor and minimal exposure to normal surrounding tissues [2-4]. SBRT is now considered the standard of care for medically inoperable early stage non-small cell lung cancer (NSCLC) based on a range of prospective and retrospective reports which demonstrate high local tumor control, low rates of treatment-related toxicity and improved overall survival when compared to conventionally fractionated radiation therapy [5-7]. SBRT can also be used in early stage operable NSCLC that refuse surgery [7], in NSCLC with local relapse after surgery or radiotherapy, in multiple synchronous and metachronous primary lung cancers, and in NSCLC with oligometastatic or oligoprogression in the lung. The role of SBRT as locally ablative therapy in NSCLC continues to expand.

Although SBRT provides excellent local control rates (98% at 3 years and 87% at 5 years) in patients with early-stage NSCLC, local recurrences may appear, most of them in the first 2 years after treatment, even up to 5 years later [6,8]. The assessment of response to SBRT is routinely done with computed tomography (CT) where the appearance of radiation-induced lung injury caused by SBRT is different from changes seen after conventional radiation therapy. The radiologist must be familiar with these changes to be able to assess response and to avoid the misclassification of benign changes as local recurrence of the tumor with the risk of unnecessary biopsies or surgery, or to avoid missing the opportunity to diagnose local relapse early when patients can benefit from salvage therapy [3,9,10]. Given that in certain cases the evaluation of the response with CT is problematic, PERCIST (positron emission tomography [PET] Response Criteria In Solid Tumors) criteria have been proposed for clinical practice as well as other 18F-fluorodeoxyglucose (^18^F-FDG) PET/CT qualitative and quantitative criteria, since it has been shown to have a high negative predictive value after SBRT for lung cancer. At present, there is no agreement on a fixed standardized uptake value (SUV) cutoff for differentiating fibrosis from local recurrence, and main guidelines only recommend ^18^F-FDG PET/CT when recurrence is suspected on serial CTs, and when ^18^F-FDG PET/CT findings suggest tumor relapse, histological confirmation is recommended in patients who are candidates for salvage therapy [11].

## 2. CT imaging findings after SBRT

Radiation induced lung changes are classified radiologically as early or late, taking into account the time interval after the completion of radiation therapy [2,12]. The early phase (within 6 months post-SBRT) corresponds to the clinical and pathologic changes of acute radiation pneumonitis, and the late phase (after 6 months post-SBRT) to chronic radiation fibrosis. Radiation induced lung injury is reported to occur in 62% of patients treated with SBRT in the acute setting and in the 91% of patients in the late setting, with the majority of patients remaining clinically asymptomatic [13].

**Figure 1:**
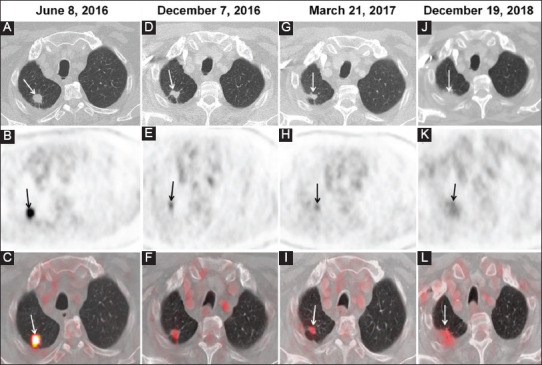
A 78-year-old male with severe COPD (chronic obstructive pulmonary disease) and domiciliary oxygen therapy. Incidental finding of lung nodule on chest X-ray. 18F-fluorodeoxyglucose (^18^F-FDG) positron emission tomography/computed tomography (PET/CT) images before stereotactic body radiation therapy (SBRT) (A, B, and C) show a solid spiculated nodule with FDG uptake and SUVmax 13,7 suspicious for primary lung cancer (arrows). The patient was treated with SBRT in October 2016. ^18^F-FDG PET/CT images in December 2016 (D, E, and F) and March 2017 (G, H, and I) showed reduction in size and lower FDG uptake (SUVmax 4.1 and 3.8, respectively) (arrows). ^18^F-FDG PET/CT in 2018 showed a subpleural atelectasis with SUVmax 3.6 (J, K, and L) (arrows).

Due to the complex beam arrangements in SBRT, the low dose regions are larger and irregular, and in contrast, the high dose area is uniform and smaller in size than in patients treated with conventional radiation therapy. This great conformality and homogeneity of the dose explain that the shape of SBRT induced lesions more precisely matches the initial treated volume and is often spherical, instead of linear (Figure 1). Reviewing the radiation planning CT is useful to interpret the images post-treatment [2,9,14].

### 2.1. Early findings after SBRT

Lung abnormalities after SBRT do not usually appear at CT before 2-3 months after the end of the treatment, with an incidence of 30% at 3 months [2,15]. The median time interval to the detection of lung attenuation changes at CT is about 17 weeks [15]. This interval is longer than for conventional radiation therapy, in which changes are seen within 4 weeks after completion of the therapy [2,10,16].

**Figure 2:**
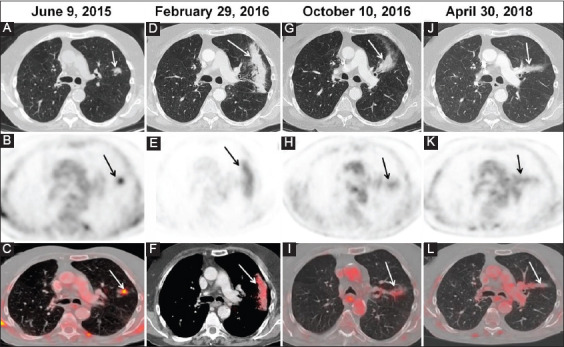
A 73-year-old male, ex-smoker, severe COPD with home oxygen therapy and right upper lobectomy in 2000 (undifferentiated large cell lung carcinoma). In June 2015, a nodule appears in the upper left lobe with histological diagnosis of squamous cell lung cancer, with FDG and SUVmax 7.3 uptake on ^18^F-fluorodeoxyglucose (^18^F-FDG) positron emission tomography/computed tomography (PET/CT) images (A, B and C). Treatment with stereotactic body radiation therapy (SBRT) ends in November 2015. Three months later, ^18^F-FDG PET/CT images (D, E, and F) show a post-SBRT pneumonitis, diffuse consolidation type, with decrease of SUVmax to 3.7 (arrows). In the ^18^F-FDG PET/CT images 11 months after the end of SBRT (G, H, and I) scar-like fibrosis is seen, stable in 2018 (J, K, and L) (arrows).

**Figure 3:**
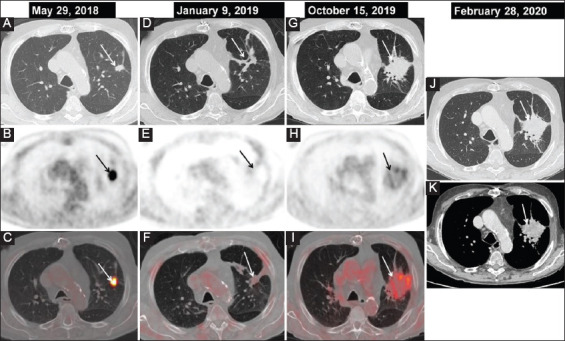
A 83-year-old male, ex-smoker, with heart transplant for ischemic cardiomyopathy with severe heart failure in 2000. Severe COPD. Stage I squamous cell carcinoma of the lung cells in the upper left lobe. 18F-fluorodeoxyglucose (^18^F-FDG) positron emission tomography/computed tomography (PET/CT) images before treatment with stereotactic body radiation therapy (SBRT) showed a solid and spiculated nodule in the upper left lobe with FDG capture and SUVmax 13.3 (A, B, and C) (arrows). Three months after SBRT the PET/CT shows changes consistent with patchy consolidation pneumonitis, associated with volume loss and decrease of the SUVmax to 2.3 (arrows). ^18^F-FDG PET/CT 12 months after SBRT (G, H, and I) shows a modification in lung changes, with rounded “mass-like” morphology and peripheral FDG uptake, with the SUV maximum of 3.5 (arrows). Image results are stable on a CT scan performed 16 months after SBRT (J and K) (arrows).

**Figure 4:**
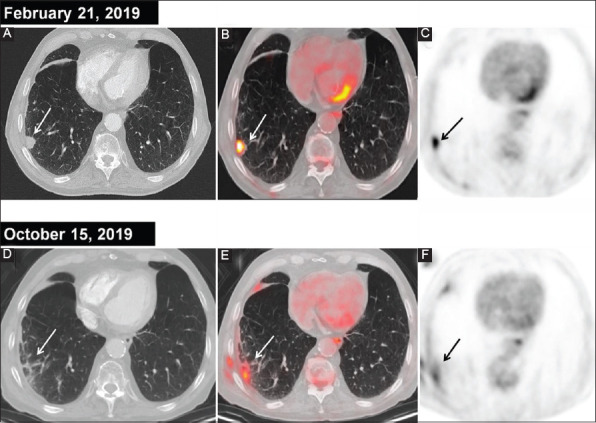
A 84-year-old male, smoker, with severe COPD, domiciliary oxygen therapy, and ischemic cardiomyopathy. He was diagnosed with a Stage I adenocarcinoma of the lung in the lower right lobe which was treated with stereotactic body radiation therapy (SBRT). The 18F-fluorodeoxyglucose (18F-FDG) positron emission tomography/computed tomography (PET/CT) images prior to SBRT (A, B, and C) showed the subpleural tumor in the right lower lobe with FDG uptake and SUVmax 8.2 (arrows). Six months after the end of SBRT, 18F-FDG PET/CT images (D, E, and F) show a ground- glass opacity and subsegmental atelectasis at the anterior lung node location (arrows).

Four CT patterns of early radiation pneumonitis have been described, following the classification by Ikezoe *et al*. (1) diffuse consolidation (Figure 2), (2) diffuse ground glass opacity, (3) patchy consolidations and ground glass opacities (Figure 3), and (4) patchy ground glass opacities [2,15,17]. In some patients, there is no increase in the density of the parenchyma in the area treated. The findings are defined as diffuse when changes exceed or are equal to 5 cm in maximum diameter or patchy if lung abnormalities do not completely fill the field of irradiation.

Lung abnormalities after SBRT are not usually seen at sites remote from the target volume. However, in patients with interstitial lung disease, especially honeycombing, radiation pneumonitis changes may be more extensive and extend beyond the radiation field [18]. Patients with emphysema, on the contrary, present lower rates of radiation pneumonitis [19]. Pleural thickening and reactive pleural effusions may occur in the first 6 months post-SBRT [2,9,12]. They are usually small, persist for months, disappear spontaneously, and do not increase spontaneously after a period of stability. When the pulmonary opacities appear before the treatment ends or beyond the radiation field, infection must be ruled out in the appropriate clinical scenario. Other findings strongly indicative of infection are cavitation and/or tree-in-bud opacities.

### 2.2. Late findings after SBRT

**Figure 5:**
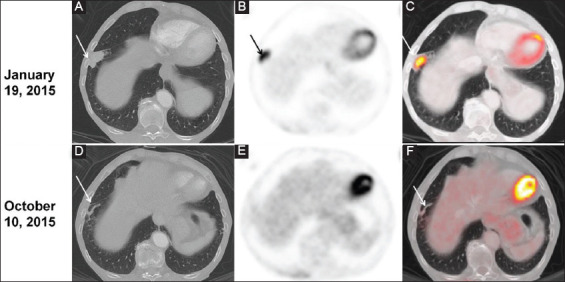
66-year-old woman, smoker, with severe COPD, and incidental finding of lower lobe nodule on chest X-ray. Axial 18F-fluorodeoxyglucose (18F-FDG) positron emission tomography/computed tomography (PET/CT) images (A, B, and C) in January 2015 showed a solid lobulated lung nodule with FDG uptake (SUVmax 12.9) (arrows), suspicious for primary pulmonary neoplasm. As the patient is not operable, she was treated with stereotactic body radiation therapy, finishing the treatment in June 2015. A 18F-FDG PET/CT in October 2015 (D, E and F) showed a linear scar without FDG uptake.

Changes at CT after 6 months post-SBRT appear in about 80% of patients and have been classified in three patterns, following Koenig classification [2,15,20]:

(1) Modified conventional: This pattern is the most frequent, can be found in about 50% of cases, and consists of a well-defined consolidation, volume loss, and traction bronchiectasis. These findings are like those seen in conventional radiation therapy, but less extensive.

(2) Mass-like fibrosis: This pattern has been reported in 7-20% of the cases. When consolidation and traction bronchiectasis are confined to a 2 cm circumferential margin around the original tumor the appearance is that of a mass-like area larger than the original tumor (Figure 3). This region corresponds to the maximal isodose curve delivered and conforms to the shape of the neoplasm treated.

(3) Scar-like fibrosis: It is a linear opacity in the region of the tumor associated with volume loss. It has been reported in 11-21% of cases (Figures 4 and 5).

Shape and location of consolidations may change with time, because fibrosis causes deformity of the lung, with displacement of the changes toward or away from the hilum [21,22]. The pattern of fibrosis post-SBRT can evolve from a modified conventional pattern to a mass-like or scar pattern. This can happen even after 24 months after therapy (which is unusual after conventional radiation therapy) and explains why the proportion of patients with a mass-like pattern of fibrosis increases after 2 years of follow-up. The mass-like pattern of fibrosis and the evolution from a modified conventional pattern to a mass-like pattern is two important imaging pitfalls that can be mistaken for tumoral recurrence (Figure 3). In addition, to these parenchymal changes, pleural thickening can be seen in more than half of patients. Sometimes pleural effusions may also develop but less frequently and usually resolve [2,12]. Features that are worrisome and suspicious for recurrent malignancy include the development of a new pleural effusion or a pleural effusion that increases in size 6 months or longer after the completion of radiation therapy or when nodular pleural thickening develops [9].

The system of classification described above has shown modest inter-rater reliability [23]. Raziee *et al*. proposed a structured radiographic reporting tool for characterization of changes post SBRT, which includes five categories: Tumor in primary site, tumor in involved lobe, consolidation, volume loss, and ground glass or interstitial changes [24]. These categories are classified as increased, stable, decreased, obscured or not present in comparison with the previous CT. However, this scale showed only fair to moderate inter-rater agreement in all categories. This is probably due to the variability in defining patterns. The current literature regarding patterns of post-SBRT radiographic changes is fragmented, partly because there is still no standardized scale to score post-SBRT fibrosis patterns across trials, and therefore the results from a given study are not immediately comparable to those from another [25,26].

Although the standard imaging modality post-SBRT is CT, the optimal follow-up of these patients remains unclear as low accuracy of Response Evaluation Criteria in Solid Tumors (RECIST, version 1.1) for predicting tumor recurrence has been reported by different authors [26-28]. In the post-SBRT setting, the main limitation is that these criteria rely on diameter alone to classify response [29]. According to RECIST, an increase of at least 20% in the longest diameter of the tumor, measured in the plane of image acquisition (axial for CT) is considered as local failure. Unfortunately, benign changes of fibrosis after SBRT may appear as enlarging opacities, especially in the case of mass-like fibrosis. Besides, the appearance of SBRT changes may be very irregular and this explains the interobserver variability in measuring non-spherical lesions. Another limitation of RECIST is the requirement that measurements be taken in the imaging plane, since craniocaudal growth may be a major predictor of recurrence and it is measured in coronal or sagittal plane.

**Figure 6:**
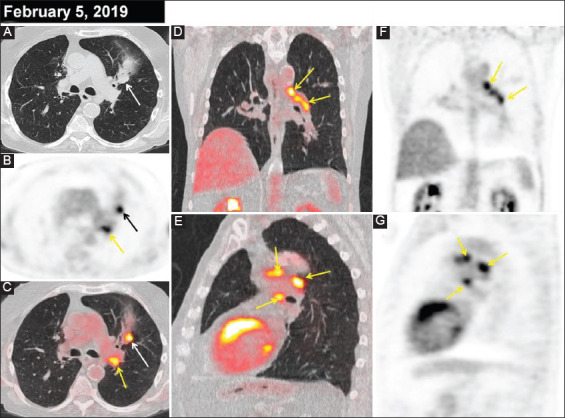
Same patient as in Figure 2. Tumor recurrence. 18F-Fludeoxyglucose (FDG) positron emission tomography/Computed tomography images in the axial (A, B, and C), coronal (D and E), and sagittal (F and G) plane show a nodule inside the scar, with FDG uptake and SUVmax 10 (white and black arrows). Left hilar and mediastinal lymphadenopathies have also appeared (yellow arrows).

Following a systematic review of the literature, Huang *et al*. identified several high-risk factors (HRF) for the detection of recurrent disease and differentiation of recurrence from radiation-induced lung injury on CT images: Enlarging opacity at the SBRT site after 12 months (Figure 6), sequential enlarging opacity, convex bulging margin, loss of lineal margin, and loss of air bronchogram (including partial loss) [13]. The same authors added a new HRF: Growth in the craniocaudal direction (>5 mm and >20%) [30]. An enlarging opacity after 12 months was the best individual predictor for recurrence, and the craniocaudal growth the second-best predictor. The presence of three or more HRFs predicted local recurrence with high sensitivity and specificity over 90% [30]. Peulen *et al*. reported that the bulging margin (Figure 6) and the craniocaudal growth are the two best predictors and, when combined, sensibility and specificity for recurrence are 85% and 100%, respectively [31]. However, these authors did not find a statistically significant association between the loss of air bronchogram and pleural effusion with recurrence. Although for several authors an enlarging opacity after 12 months is highly suggestive of recurrence [25,32], other authors do not consider this finding useful as it is frequently found in patients without recurrence (Figure 3) [33,34]. For Halpenny *et al.*, a new bulging margin is the only significant predictor for recurrence [35]. Other studies have insisted in that current criteria of interpretation of CT images are insufficiently accurate for clinical use in the detection of local recurrence, because HRFs may be found in at least 50% of patients without recurrence and three or more HRFs can be found in 25% of patients without recurrent disease [33,35,36].

Despite the mentioned limitations, the seven HRFs may be used in an algorithm proposed by Huang *et al*. to help in the adequate follow-up of these patients, preferably in a multidisciplinary team discussion [30,37]. Low risk patients without HRFs can be followed with CT; intermediate risk patient with one or two HRFs can undergo ^18^F-FDG PET/CT or close follow-up with CT at 3 moths and high risk patients with three or more HRFs could proceed to biopsy or salvage therapy; ^18^F-FDG PET/CT could also be considered [3]. Although Takeda *et al*. reported that ^18^F-FDG PET/CT could help to detect local recurrences with greater accuracy, CT is still the first-line modality to serve this purpose, and ^18^F-FDG PET/CT is only performed when recurrence is suspected. Biopsy and/or surgical or nonsurgical salvage therapy can be considered if safe and when investigations are non-reassuring.

Recently, a Delphi consensus process by international opinion leaders in thoracic radiation oncology and radiology concluded that the findings suggestive of a local recurrence on CT scan were as follows: Infiltration into adjacent structures, bulging margins, sustained growth, mass-like growth, spherical growth, craniocaudal growth, and loss of air bronchograms [38]. The Delphi consensus recommended use of ^18^F-FDG PET/CT scans only when local recurrence was suspected.

In 2018, international expert opinions provided the following consensus statements for the follow-up of patients with early-stage NSCLC treated with SBRT [38-40]:

Follow-up imaging modalities

1. Thoracic CT scans should be used as a part of routine imaging follow-up.
2. The data informing the use of FDG-PET/CT scans in the follow-up setting is limited.

Until further evidence is available, we recommend the judicious use of FDG-PET/CT scans and note that they are currently not included in routine imaging follow-up at most of the institutions represented by the consensus panel.

If there is a suspicion for recurrence, FDG-PET/CT scans are strongly recommended.

Frequency of follow-up imaging

1. In the 1^st^ year of follow-up, thoracic CT scans are recommended at 3, 6, and 12 months after SABR.
2. In the 1^st^ year of follow-up, thoracic CT scans are not recommended at 6 weeks after SABR.
3. In the 2^nd^ year of follow-up, thoracic CT scans are recommended at 18 and 24 months after SABR.
4. In years 3 through 5 of follow-up, thoracic CT scans are recommended annually.
5. After 5 years, thoracic CT scans are still recommended; however, there is no consensus regarding the frequency with which they should be ordered.

Detecting local recurrence on follow-up CT imaging

1. RECIST 1.1 criteria are not sufficient for detecting recurrence in routine CT imaging follow-up.
2. A formal scoring system should be used to classify high-risk imaging features suggestive/predictive of local recurrence following SABR.
3. Previously published and validated high-risk features on CT imaging should be used as a formal scoring system.
4. The following CT findings should increase suspicion for a local recurrence:
  - Infiltration into adjacent organs/structures
  - Sustained growth over serial scans
  - Bulging margins
  - Mass-like growth
  - Predominantly spherical growth
  - Craniocaudal growth
  - Air space obliteration/loss of air bronchograms.

Salvage treatment for local recurrence

1. Local salvage therapy without pathology is acceptable if imaging findings are highly suspicious and a biopsy is not safe or feasible.
2. Local salvage therapy without pathology is acceptable if imaging findings are highly suspicious and a biopsy has been attempted but was non-diagnostic.

*CT: Computed tomography; FDG-PET: Fludeoxyglucose positron emission tomography; RECIST: Response evaluation criteria in solid tumors; SABR: Stereotactic ablative radiation therapy*.

Both National Comprehensive Cancer Network and ESMO guidelines agree that the most appropriate follow-up strategy for NSCLC after SBRT treatment is to perform a chest CT every 3-6 months during the first 2 years and annually in the following 3 years [41,42]. However, as mentioned previously, SBRT frequently produces pneumonitis in the treated lung, with radiological changes on the control CT performed at 2-3 months after treatment, which can either resolve or evolve to fibrosis [34]. These changes are sometimes indistinguishable from the presence of residual tumor on CT. For this reason, the above-mentioned guidelines give an important value to the metabolic information provided by PET, recommending the selective use of ^18^F-FDG PET/CT (performed together with a diagnostic CT with contrast enhance if possible) when recurrence after SBRT is suspected on serial spiral chest CT (IIIB level of evidence) [41,42].

## 3. ^18^F-FDG PET/CT imaging findings after SBRT

The use of ^18^F-FDG PET/CT in routine monitoring of patients treated with SBRT is a controversial issue, and most experts still consider it as just an optional tool [11]. Some limitations of PET-CT compared to CT include cost, radiation dose, and availability. Resolution is also sometimes lower than that of CT, especially if the CT scan of PET-CT is performed in expiration or when studying small lesions. In PET studies false positives secondary to infection should be considered and correlated with the CT study.

Nevertheless, it is well known that metabolic information provided by ^18^F-FDG PET/CT has an additional value over CT in the assessment of response to therapy in patients with locally advanced lung cancer as changes in SUV treatment seem to be associated with tumor response and survival [43-49]. PET-CT also helps to guide biopsy when the CT images are confounding and are superior to CT for detection of regional and distant metastases.

In early stage lung cancer treated with SBRT, SUVmax measured in the primary lesions has demonstrated to have prognostic relevance concerning outcome and local control rates and to provide important information for patients receiving SBRT [50,51]. Furthermore, one of the more significant benefits of PET compared to structural imaging techniques is that metabolic changes tend to take place earlier than structural modifications. However, the best way to assess the response to treatment is not established.

In 1999, the European Organization for Research and Treatment of Cancer (EORTC) proposed some criteria to assess tumor response to therapy, based on ^18^F-FDG uptake as a metabolic response biomarker. Four degrees of metabolic response are distinguished: Complete response, partial response, stable disease, and disease progression [52]. These criteria recognize that subclinical metabolic changes can be observed early by PET with or without morphological correlation, meaning that tumors response or progression can be detected not only because of size changes but also when SUV values decrease or increase.

The PERCIST1.0 (PET Response Criteria In Solid Tumors) criteria [53] for the assessment of the response in solid tumors were proposed by Wahl in 2009. This document aims to provide useful response criteria for clinical practice and it is actually used in clinical trials that investigate the role of SBRT [54]. PERCIST criteria are based on a combination of the radiologic RECIST and the EORTC criteria and according to it, the response to therapy is measured as the percentage of the change in glucose uptake: Metabolic disappearance of the active tumor is considered a complete metabolic response, whereas a 30% decline in peak SUV comparing the most intense lesion before treatment and the most intense after treatment is considered a partial response.

Some authors reported that PERCIST-based response to treatment correlate with progression-free survival and loco regional control in patients treated with SBRT for early-stage NSCLC [55], while others defend that a meticulous visual assessment of ^18^F-FDG PET/CT images is of most importance in this setting [56], but there are not yet robust metabolic criteria for evaluating lung patients treated with SBRT.

The high negative predictive value of ^18^F-FDG PET/CT in follow-up after SBRT has been highlighted in several studies [57,58]. However, some authors have reported that ^18^F-FDG PET/CT performed early after treatment may be an insensitive test for the evaluation of recurrence as the inflammation involved in radiation pneumonitis can falsely elevate SUV within and around the treated tumor bed, what can be translated into a sensitivity decrease of the technique [59]. This falsely elevated SUV might be in relation to the accumulation of FDG in macrophages present in post-treatment inflammatory processes and can be found up to 1 year or even 2 years after treatment without later evidence of clinical recurrence [60,61].

In the last years, more authors are supporting the idea of the usefulness of ^18^F-FDG PET/CT in the follow-up of lung cancer treated with SBRT, always emphasizing the importance of a carefully interpretation of radiotracer uptake depending on the time of acquisition and the clinical context of each patient. Tyran *et al*. observed in their study that the metabolic response after SBRT occurs within the first 3-6 months after treatment, while late changes that can be evidenced up to 2 years and are usually seen as low metabolic activity [62]. Ding *et al*. also demonstrated in their study that a ^18^F-FDG PET/CT performed 3 months after treatment can assess tumor response fairly accurately, using as complete response criteria a post-treatment SUV_max_ ≤1.9 [63]. In the same way, Bollineni *et al*. found in their series of 132 patients that a SUV_max_ >5.0 at 12 weeks after SBRT might indicate an increased risk of local failure and the need of a closer monitoring of these patients, especially those suitable of at least sublobar resection [51]. In addition, Sheikhbahaei *et al*. analyzed multiple studies showing that ^18^F-FDG PET/CT performed early after therapy not only predicted response but also resulted in the initiation of a new treatment plan [64]. Furthermore, multiple authors have reported that not only SUV_max_ after SBRT but also the relative reduction of early post-treatment values with respect to pre-treatment ones could predict outcome (local control and overall survival) [49,62,65,66]. The possibility of performing a dual-phase ^18^F-FDG PET/CT with early and delayed PET images is under investigation, based on the idea that tumor FDG uptake continues to increase over the time for hours, while this does not usually occur in areas of inflammation [67,68]. However, this proposal remains still unclear and challenging to implement into clinical practice for a matter of cost and time.

Despite the usefulness of SUV_max_ for monitoring the tumor response to treatment, there are some considerations to be aware of. For example, tumors of small size or those located in areas of physiological movement can lead to underestimated SUV_max_ values and tumors with central necrosis may show little change in SUV_max_ over time [69]. For this reason, other parameters such as metabolic tumor volume (MTV) or glycolysis rate (Total lesion glycolysis [TLG]) have been developed, to integrate the information of metabolic activity within the tumor volume. However, evidence in the literature describing the value of these parameters is scarce [64].

## 4. Future Directions

### 4.1. Radiomics

Due to the limitations of the qualitative CT criteria used to detect recurrent disease after SBRT, there is an active search of accurate and standardized quantitative CT measures. As some of the tumor behavior information is contained in medical images but is not often appreciable by human eye, mathematical techniques have been developed in the field of radiomics, allowing the extraction of different imaging biomarkers with predictive value to later analyze them using bio-informatic systems. Radiomics image features analyzed through mathematical techniques describe the gray level patterns of an image: First-order statistics (distribution of intensity histograms), second-order texture features, and size and shape based features (sphericity, spiculation, and roughness) [29,70]. Using radiomic image features in areas of CT changes post-SBRT, Mattone *et al*. could predict recurrence as early as 6 months after SBRT before it can be differentiated by the human eye, and with higher prediction accuracy [71]. Texture analysis has also been used in CT and in ^18^F-FDG PET/CT to predict the response to SBRT of patients with early stage NSCLC and to quantify radiation-induced lung damage [72-76].

Just as in CT, radiomic features on PET can be classified into first, second, and high order. First-order radiomic features are those standard parameters which do not provide any spatial information, including SUV_max_, VMT, and TLG [69,77-81]. Despite their promising results, efforts in radiomics are currently focused on second- and high-order statistical features which can provide spatial information and can measure the tumor heterogeneity. These features are also called “texture analysis” and seem to be related to greater tumor aggressiveness and worse response to treatment [82]. Although some of these features such as entropy or dissimilarity have already demonstrated its predictive value in patients with NSCLC treated with SBRT [74,83,84], others are still being tested and acquisition protocols and segmentation tools must be standardized and validated before incorporating them into clinical practice.

### 4.2. New treatment approaches: Immunotherapy and lung SBRT

There is a significant interest in approaches that combine RT with immunotherapy to improve systemic control in NSCLC due to SBRT can modulate the host immune system in the local tumor microenvironment and to activate a tumor-directed systemic immune response. However, it is not clear how best evaluate the response to this combined treatment as immune-related response criteria have been developed, but its value to evaluate response after combined SBRT and immunotherapy should be tested [85]. The contribution of ^18^F-FDG PET/CT in monitoring NSCLC treated with immunotherapy has been studied, and some metabolic criteria that could have a better prognostic value in the assessment of tumor response than current morphological criteria have been described [86]; therefore, ^18^F-FDG PET/CT could have a role on implementation of SBRT combined with immunotherapy. However, prospective studies supporting the usefulness of ^18^F-FDG PET/CT in the follow-up of this combined therapeutic technique are needed to endorse its use in clinical practice.

## 5. Discussion

The assessment of response after lung SBRT has major limitations and there is a need to establish morphological and functional response criteria that can guide clinical decisions. In this article, we have reviewed the morphologic and metabolic findings on CT and ^18^F-FDG PET/CT after lung SBRT, highlighting the difficulties in assessing early response and the limitations in unifying response criteria. The absence of robust response criteria has direct implications not only for disease management but also for clinical trials since treatment efficacy is evaluated according to different criteria and therefore outcomes cannot be compared.

The limitations of CT in this setting are well known: Suspected signs of relapse or progression are controversial, and some changes must occur over many months to be considered suspicious, which excludes the possibility of a confirmed response within a few months of treatment. A high rate of complete responses in the first 2 years after SBRT treatment is described in the literature. However, size changes usually take time to occur and the radiological changes that appear in the adjacent lung parenchyma from the 3^rd^ to 6^th^ months make it difficult to measure tumor size and hence to assess response. This makes us think that may exist differences between researchers when evaluating the response to treatment during the 1^st^ year of follow-up, since in few patients the tumor will disappear and yet they may have a complete response (with residual fibrosis). The high rate of complete response reported in the literature suggests that different radiological criteria may have been used.

CT is still considered the test of choice for assessing response to lung SBRT, although there is a clear role of ^18^F-FDG PET/CT for the differential diagnosis of fibrosis against relapse or progression due to its high negative predictive value, and more and more studies point to the relationship of the SUVmax at 2 or 3 months after treatment with the outcome. Furthermore, several studies point out that the metabolic response is earlier than the morphological response, but its prognostic value has not been clarified. The main reason for 18F-FDG PET/CT not to be considered the test of choice for follow-up is because FDG uptake in areas of pneumonitis limits the assessment of the treated tumor and causes false positive results. In addition, the lack of unified criteria for assessing metabolic response is also notable and there is no agreement on the cutoff point to be considered.

In light of these difficulties, further studies are needed to clarify the usefulness of assessing the metabolic response in early follow-up (perhaps with new tracers, associating magnetic resonance imaging, dual-PET, etc.) to determine the response to treatment before changes of pneumonitis prevent its correct assessment. In addition, regional or distant relapses would be assessed more sensitively than by CT, and the chances of offering rescue treatment when the tumor has not yet spread would be greater.

The limitations of this review are significant, as it is not a systematic review and the studies mentioned include few patients and different methodologies. In addition, series of patients treated with lung SBRT often include patients without a histological diagnosis or molecular study. Metabolic and morphological response could be different for different types of tumors and studies with more patients are needed to find out if there are different response patterns depending on the histology and molecular study.

## 6. Conclusions

Imaging plays a crucial role in the follow-up of patients treated with SBRT and it often determines the next steps in a patient’s clinical management. Although the standard imaging modality post-SBRT is CT, anatomic criteria have been shown to have limitations in this setting and in patients with fibrotic changes the use of a combination of high-risk features and PET/CT findings, should be considered. The main guidelines do not consider routine 18F-FDG PET/CT for follow-up, mainly because post-SBRT inflammation causes false positives, but there is increasing evidence in the literature of its potential value for monitoring response after lung SBRT.

Future challenges in imaging after SBRT include the standardization of response criteria in CT and PET imaging, radiomic analysis, and to determine optimal way to measure response to combined treatment with immunotherapy and SBRT in lung cancer.
